# Bipolar disorder and sexuality: a preliminary qualitative pilot study

**DOI:** 10.1186/s40345-023-00285-9

**Published:** 2023-02-03

**Authors:** Helle B. Krogh, Maj Vinberg, Gitte Lee Mortensen, Ditte Skakke, Dorthe Nielsen, Annamaria Giraldi

**Affiliations:** 1 Sexological Clinic, Mental Health Center Copenhagen, Copenhagen University Hospital, Ole Maaloes 14, 2100 Copenhagen, Denmark; 2 Mental Health Center, Northern Zealand, Copenhagen University Hospital, Mental Health Services CPH, Hillerød, Denmark; 3 Copenhagen Affective Disorder Research Center (CADIC), Psychiatric Center Copenhagen, Copenhagen, Denmark; 4 AnthroConsult, Aarhus, Denmark; 5 Odense University Hospital, Odense, Denmark; 6 Department of Clinical Medicine, University of Copenhagen, Copenhagen, Denmark

**Keywords:** Bipolar disorder, Sexuality, Sexual function, Sexual dysfunction, Hypersexuality, Qualitative research

## Abstract

**Background:**

Individuals with mental health disorders have a higher risk of sexual problems impacting intimate relations and quality of life. For individuals with bipolar disorder (BD) the mood shifts might to a particular degree affect their sexual function with possible hypersexual interest during manic episodes and low sexual interest during depressive episodes. The diagnosis is often given in late adolescence, which may impact sexual identity and development. Only a few studies have looked at BD and sexual life, with no qualitative research on the topic.

We conducted a qualitative pilot study exploring sexuality in connection to mood swings in five participants with BD.

**Results:**

Thematic content analysis revealed five themes: (1) sexual drive and impulses, (2) sexual behavior, (3) thoughts and feelings in relation to sexual issues, (4) intimate relationships, and (5) sexuality and identity. During manic episodes the participants described having a higher sexual drive, leading for some to more sexual interactions. During depressed episodes, the sexual drive in the three female participants was low, however, in the two men, rather than a reduced sexual drive, a more self-destructive way of engaging in sex prevailed. The sexual outgoing behavior during manic phases was described as joyful, with no feelings of shame connected to it. However, the shifts in sexual drive connected to mood shifts affected the participants’ relationships negatively. Further, all the participants described having outgoing sexual behavior in their youth.

**Conclusions:**

Overall, changes in sexual drive may act as a trigger or early warning symptoms of new episodes, pinpointing the clinical relevance of addressing sexuality in individuals with BD. In general, sexual drive followed affective episodes. However, during depressive episodes sex could be, instead of reduced drive, associated with negative feelings.

All participants described having an outgoing sexual behavior in their youth before the onset of BD, which might be essential to consider if there is a clinical suspension of BD in an individual.

## Background

Bipolar disorder (BD) is a severe psychiatric disorder, characterized by two or more episodes with either hypomania or mania, depression or mixed episodes, and with euthymic periods in between (American Psychiatric Association 2014). BD is one of the most important causes of disability worldwide, (Merikangas et al. 2011; Steel et al. 2014) leading to both cognitive and functional impairment with a substantial impact on social and working life (Ferrari et al. 2016). BD often has an onset in late adolescence and early adulthood, coinciding with the development of sexual identity (McMillan et al. 2017). Healthy sexual development is important for gaining autonomy in one’s sexual decision-making and healthy sexual relations, which contributes to enhancing the quality of life (Koyama et al. 2009; Quinn and Browne 2009).

Sexuality should be viewed from a biopsychosocial perspective and describes sexual thoughts, feelings, behaviors and how (if) you experience sexual attraction (WHO 2002). Sexuality also covers sexual function, which is defined as the sexual response phases: desire, arousal (in men erection and in women lubrication), orgasm, and resolution. Sexual dysfunctions are defined as problems with these phases as well as sexual pain and vaginismus, preventing the person from having a satisfying sexual activity and being distressed by the problem (McCabe et al. 2016). In people with mental health problems, sexual dysfunction is more prevalent than in the background population and constitutes an underestimated problem across all mental health diagnoses (Zemishlany and Weizman 2008; Dell’Osso et al. 2009; Waldinger 2015). The increased risk of sexual dysfunction in individuals with mental disorders may be due to the use of psychotropics or to the disorder itself (McMillan et al. 2017; Namli et al. 2018; McCabe et al. 2016). In affective disorders, the impact of depression on sexual function is well described, (Zemishlany and Weizman 2008; Williams and Reynolds 2006; Phillips and Slaughter 2000) whereas research on how BD affects sexual life is sparse. An Italian study by Dell’Osso et al. from 2009 investigated the occurrence of sexual dysfunctions in 60 men and women with BD compared to 82 men and women with unipolar depression (UD) and 101 healthy controls (Dell’Osso et al. 2009). They showed that sexual dysfunctions are significantly more common in individuals with BD and UD compared to healthy individuals. Furthermore individuals with BD reported significantly increased interest in sex and a frequent change of sexual partner compared to UD and healthy controls (Dell’Osso et al. 2009). A previous study from our group compared 61 women with BD to data from 429 healthy controls on sexual impairment and sexual distress. This study revealed that women with BD did not have a significantly higher prevalence of impaired sexual function or sexual dysfunction, but compared to the background population they reported to be significantly more sexually distressed (Sørensen et al. 2017).

Marked sexual energy or sexual indiscretions are included in the diagnostic criteria for BD in the International Classifications of Diseases 10 (ICD 10) (WHO 1993) and Diagnostic and Statistical Manual of Mental Disorders V (DSM-V) (American Psychiatric Association 2014) and the symptoms are well described clinically. However, the literature on hypersexuality is sparse. A review from 2016 (Kopeykina et al. 2016) found only three studies, two cross-sectional and one case report, representing recent literature describing hypersexuality and BD. The review revealed that during mania, individuals with BD have more risky sexual behavior compared to other psychiatric patients. It was further described that hypersexuality in manic episodes and hyposexuality in depressive episodes affect partners’ sexual satisfaction in a negative way, and this often persists into inter-episode periods (Kopeykina et al. 2016). In general BD was associated with problems establishing and maintaining couple relationships (Kopeykina et al. 2016).

Health care providers do often not address sexual issues and patients do not spontaneously bring the subject up, implying an underestimated and perhaps tabooed problem in clinical practice (Quinn and Browne 2009). Improved understanding of how sexual health and function in individuals with BD may be related to their disease and everyday life, including relationships, might help to address these issues in clinical practice and improve the quality of life and sexual well-being in patients with BD.

To our knowledge, no qualitative research about the subject has been published and there is a need for clinical studies investigating the experiences of individuals with BD of the impact of BD on their sexual health. Therefore, this pilot study aimed to produce in-depth knowledge from a qualitative examination of individuals with BD on the possible interactions between BD and sexuality and sexual function. Further, we wanted to explore whether individuals with BD would feel ashamed about their sexual behavior, thoughts and/or initiatives during their manic episodes, and whether potential hypersexual function can have unintended and serious implications that potentially worsen the depressed episodes. Finally, we aimed to elucidate whether the participants found it relevant to implement sexological treatment in addition to their usual counseling.

## Methods

### Participants

Qualitative interviews were carried out with five patients (three women and two men) with a diagnosis of BD, according to ICD-10, in May to June 2019. The patients were included from an outpatient clinic, the Copenhagen Affective Disorder Clinic, Psychiatric Center Copenhagen, where all of them were attending a 2-year combined medical and psychological treatment, including an 18-week psychoeducation group. All the participants were in remission or partly remission during the time of the interview. When arriving at the clinic for their regular appointments, the individuals received oral and written study information and an invitation to participate. They gave permission for the obtaining of basic demographic data and access to their medical journal, and participation had no impact on the course of treatment. There were no exclusion criteria regarding the type of medicine, relationship status, type of BD, sexual orientation, gender, age, or time since diagnosis for inclusion in the study. The study was carried out in accordance with the GDPR and did not require Danish ethical committee approval. It was registered and approved by the Capital Region’s Knowledge Center for Data Reviews (Journal-Nr.: P-2021-172).

### Qualitative interviews

A literature search was carried out regarding BD and sexual function and formed the basis for the design of a semi-structured interview guide. The interviews were carried out by author (DS). The interview started with questions about the participants’ demographics followed by descriptions of the participants’ sexuality. The interview then addressed the participants’ thoughts about their sexuality being affected by their BD and vice versa, how sexual problems, if any, were handled, and whether they affected their relationship. Finally, the need for including sexual and relationship issues in the clinical practice was discussed.

### Analyses

The interviews were recorded and transcribed verbatim by DS and analyzed thematically by two of the authors (GLM and HBK) using thematic content analysis (TCA), which is a descriptive presentation of qualitative data (Anderson 2007). Initially, the recordings were listened to and the transcripts were read and then re-read. Then the interviews were coded (categorized) separately by GLM and HBK into topics that were raised during the interview. The categories were then discussed, some collapsed, and some split up in sub-categories. Following this, the categories were thematized. Finally, the connections between themes and topics were analyzed, e.g., how specific moods might be related to certain sexual impulses or behavior, and how this might affect relationships and well-being. An analysis of the terminology used to speak about the subject and the impact of the interviewer/interviewee interaction was also carried out. Author GLM produced a report describing the findings from the qualitative interviews, including the methodological and analytical steps. This was presented for and discussed with the rest of the author group. The citations in this paper were translated from Danish into English by HBK and approved by all authors.

## Results

### Participants’ characteristics

The participants’ ages varied from 25 to 46 years. The time since the participants’ diagnosis with BD varied from 0.5 to 22 years before the interviews. Three had a diagnosis of BD type 1. One participant was studying, three were working full time, and one was in subsidized part-time employment. One of the women and one of the men were in a relationship, living together with their partner. Four participants were heterosexual, and one homosexual. All patients were treated with lamotrigine (200–250 mg/day), four were additionally treated with low dose quetiapine (100–250 mg/day) and one was also prescribed lithium (24 mmol/day).

### Five main themes

**Fig. 1:**
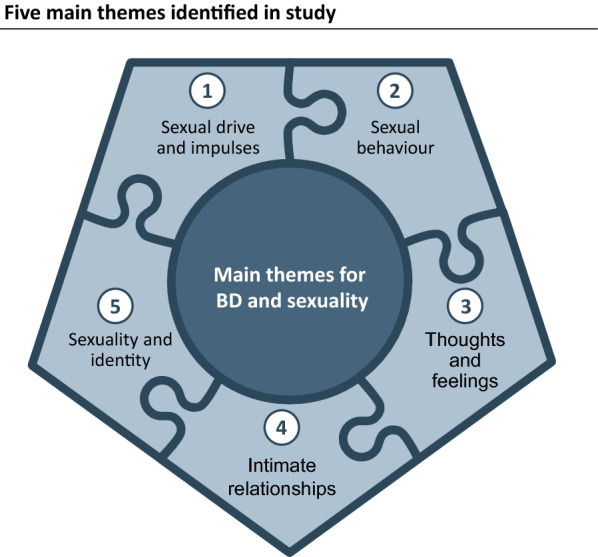
Main themes on BD and sexuality

The analysis identified five main themes regarding the participants’ perspectives on their BD and sexuality: (1) sexual drive and impulses, (2) sexual behavior, (3) thoughts and feelings in relation to sexual issues, (4) intimate relationships, and (5) the connection between sexuality and identity (Fig. 1). As the participants’ sex drives (lust or urge) did not necessarily lead to actual behavior, these two themes should be distinguished. In addition, sexual behavior (acts) was not necessarily related to pleasure, positive thoughts, or relationships, which made it relevant to distinguish between these themes as well. Finally, the participants’ sexual issues were recurrently linked to self-perceptions, i.e., identity issues.

### Theme 1: sexual drive and impulses

All participants described having always had a high sex drive compared to friends and sexual partners, also before the first episode of hypomania or mania. In general, their sexuality changed depending on the affective phase they were in. When the participants were hypomanic or manic, they tended to have a higher sex drive, to desire more experimental sex, and they were easily sexually bored. Several participants described their higher sex drive not only as a lust but as a *need* or kind of *internal pressure/urge* to have sex. One of the women described the following:

*“But it’s also one of the symptoms I’ve noticed (increased sex drive when it’s on its way (mania). Because I'm not always good at noticing it, and then I don’t realize it until I'm in the middle of it. And it is also something that I discover like this; “Wow, I seem to be thinking about it a lot at the moment, and I really need to masturbate.” And then I think: “Oh, yes, maybe I'm in that phase right now.”*

During depressed states, the participants typically described having a low sex drive. Yet for the two men, depressed episodes also implied a more self-destructive way of engaging in sexual relations and negative feelings related to sex rather than just a lowered overall sexual impulse.

*“When I have mood swings, my sexuality also swings enormously. So, when I'm manic, I want to shag anything I see, almost. And when I'm depressed, I sometimes want to have sex with people, but then it's in a slightly nastier way; I mean people have to be a bit nasty to me. Otherwise, it has to be something I don't need to get emotionally involved in at all.”*

### Theme 2: sexual behavior

Overall, the participants described a wild youth, characterized by restlessness, difficulties in finding out what they wanted in life, and frequent shifts in interests. With respect to their sexual lives, four participants described chaotic relations, many partners, and a sexually experimental behavior sometimes exceeding one’s own and others’ boundaries. Alcohol was often involved in sex, and relations were affected by infidelity. The three women described that they had been more sexually outgoing than their peers from an early age. They described instances where their sexual behavior had been considered inappropriate, e.g., excessive flirting and acting out sexually in front of friends’ parents or at school reunions. One participant had had three abortions when she was 14–19 years old.

*“There was, for example, one time when I danced with all their parents and danced with everyone, and I was just all over the place. And then I felt sick and a little tired, and I had been lying with one breast sticking out of my dress. Then my friends were like: "Now that's enough. Now we're taking you home”.”*

The two participants who were in relationships at the time of the interview still tended to flirt with others when they were in hypomanic or manic phases, and the way they had sex with their partner changed with the phases of the BD. The woman described how she might wake her partner up in the middle of the night to have sex and suggested having more experimental sex than the partner wanted. For the participants who were single, there were differences in how their sexual behavior changed during hypomanic and maniac phases. The single male had more sexual partners during his manic episodes, while the two single women masturbated more often, but did not seek sexual partners. Instead, they described having learned to control their high sex drive and using their energy in other ways, e.g., creatively or with projects, which they considered a way to take care of themselves and avoid negative intimate experiences. One of the women explained that with age she had learned that relationships and sexual relations tended to destabilize her disease.

*“My horniness is turned on and off depending on whether I'm manic or not. The difference is that I’m not in a relationship or several anymore. It (the horniness) has not subsided. Now I'm adult enough to have renounced it because it was so complicated. (It was a) strange combination of a lack of self-esteem and overestimating myself.”*

During depressive episodes, the participants’ decreased sex drive mainly affected those who were in a relationship. The woman described that her partner might feel rejected, and the lack of intimacy created a distance between them. The other two women who were single were simply not orientated towards sex during these phases. Here, a gender difference was observed since both men described that depression might not be associated with reduced sex drive but rather with a negative sex drive that was self-destructive or that the intimacy became frustrating or saddening due to a lack of sexual and emotional satisfaction.

### Theme 3: thoughts and feelings about sexuality

**Fig. 2:**
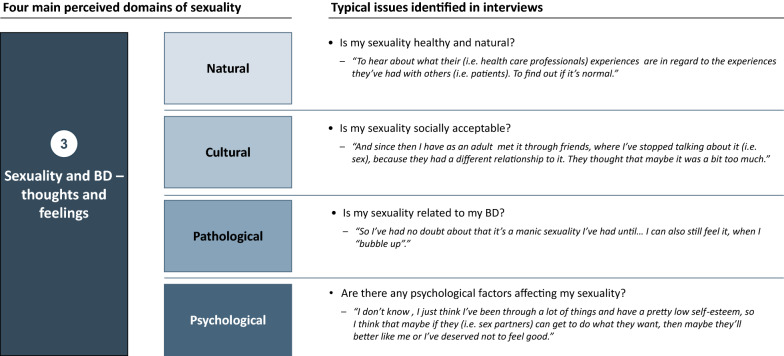
Perceived domains of sexuality based on the qualitative interviews with individuals with BD

Across the interviews, the participants speculated whether their sexuality might be related to their BD (i.e., if it was pathologic), whether it was “natural” (a physiological drive and need), “normal” (i.e., in accordance with cultural and societal norms), and/or related to psychological factors (e.g., earlier life experiences) (Fig. 2).

The participants’ distinctions between pathological, natural, socio-cultural, and psychological factors were related to their views on their own sexuality, including feelings of shame, responsibility, and the means to control their sexual drive. As such, these perceptions were connected to the participants’ coping strategies and thoughts about sexological treatment. Some participants wanted to know if their sexuality was part of their BD, while others speculated whether their childhood experiences had a negative psychological effect on their intimate issues and adult relationships. The participants’ views on their sexuality could thus be linked to a wider self-perception that was not necessarily related to their BD.

When it came to changes in sex drive following mood phases, the participants described that a high sex drive/urge was not necessarily connected to a pleasurable feeling, nor was having intercourse. One of the women explained that it was not always lust that made her have sex, despite having had several sexual affairs and flirting and being sexually curious. Her satisfaction mainly relied on getting men to long for her. One of the men also described never actually reaching sexual satisfaction, even during intercourse, no matter for how long or how he had sex. He always longed for more, which might lead to a sense of desperate insatiability.

In general, the participants did not consider the number of their sex partners and extent of sexual experiences to be problematic. They did not feel ashamed, although they were aware that others might look down upon their behavior.

*“I wasn’t ashamed like that when I was depressed, I mean, I didn’t start brooding over what I'd done. But I've sometimes felt ashamed briefly, because when I'm at my most manic, I'm too crazy about them and I don’t notice their signals that it hurts their knee or something. When I might be a bit too abusive. So, it was really embarrassing if I met them again. But it hasn't bothered me to the point that I've in general felt that my sexuality is too much… and over time, I've acknowledged it and become more considerate. (…) In reality, I have a very unproblematic relationship with my sex life. It’s more the emotionel part that’s difficult.”*

### Theme 4: intimate relationships

Several participants mentioned having a history of intense love affairs, short-lived, chaotic relations, and confusion about the connection between love and sex. Some reflected on their search for love and attention through sex, about uneven relations, or people who only wanted them for sex.

The participants were especially concerned with how changes in their sex drive affected their love life. One of the men described problems with engaging in close relations and a self-destructive sexual behavior that prevented him from having healthy romantic relationships. The other man’s insatiable need for sex and desire was a constant conflict in his marriage and the reason why the couple could “never meet or be in the same place.” One woman described that the changes in her sexual desire affected her relationship, making her worry about whether her boyfriend would leave her and whether they would be able to have kids. The two single women felt that rather than having sexual problems, more importantly, they had difficulties with emotions and entering and maintaining romantic relationships, and one woman described how heartaches affected her BD.

*“I mean, my psychiatrist knows when I’ve had these love affairs and what they lead to in terms of mood swings. When it happens, I often become manic because I turn it around from short-term depression for three days. Then I gather myself around something and go all the way down. But then I also drag myself up again, and then it becomes a sort of manic phase when I kind of do anything to forget it and keep going. But then I keep going too much. And then I stop sleeping and start rushing around.”*

### Theme 5: the connection between sexuality and identity

For all participants, their sexuality was closely related to their identity. For two of the women, it had been a positive part of their self-perception to be sexually experienced and adventurous. One described that her sexuality had overruled negative thoughts and low self-esteem and that her teenage friends had had more complicated body images. When the women were young, being the sexually outgoing and experienced girl among their peers was part of their identity and role. One of the men said he had always attracted people, also without being aware of it, which he linked to a certain enticing energy that comes with being bipolar.

*“It was connected to a big lack of self-esteem too where I thought things about my body and I was too fat, but somehow my sexuality just overruled those thoughts. I didn't lie there and think about whether my stomach was too fat at all once we’d got started.” (woman).*

### Participants’ thoughts about sexual counseling

In the final part of the interview the participants were asked if they felt sexual issues should be part of their treatment and conversations with their psychiatrist or other health care professionals. The participants’ wishes for sexological treatment were especially linked to three different aspects: (1) whether they considered their sexuality to be problematic, (2) whether they thought the issues with their sexuality could be treated, and (3) whether they were already talking with their health care provider about sexual issues or felt they could do so. In general, these participants did not call for specific sexological counseling, either because they did not see their sexuality as problematic or because they already felt they could talk with their psychiatrist about it. One participant was depressed at the time of the interview and stated that she could not see herself talking about sexual issues when she was in this state.

### Main factors affecting sexuality issues

**Fig. 3:**
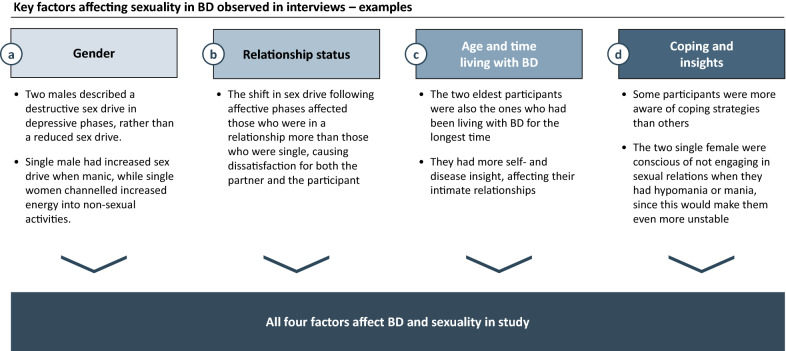
Key factors affecting sexuality in BD

Across the major themes described above, the analysis of the interviews pointed to several factors influencing the participants’ experiences with their sexuality, including (a) their gender, (b) their relationship status, (c) their age and time of diagnosis, and (d) coping strategies and insight (Fig. 3).

The interviews suggested that there may be gender differences (Fig. 3) (a) in the way the participants viewed their own sexuality. The men described a negative sex drive in depressed episodes, rather than merely a lower sexual drive, which was more common in the women. Among the participants who were single, there also seemed to be a gender difference. The single man sought more sexual partners during hypomanic or manic episodes; the two women masturbated more than usual but otherwise channeled their energy towards other activities.

Being in a relationship or not (b) was important to the consequences of the participants’ sexual drive and behavior. Those in a relationship had difficulties being on the same level as their partner, which created dissatisfaction to both, even in euthymic phases. Those who were single longed for serious romantic relationships, rather than just sex. One of the single women said that she never engaged in one-night stands because they made her feel empty and hollow. The single male considered his high sexual activity level as really being a quest for love, but instead this brought him to self-loathing and humiliation.

With age and time since the BD diagnosis (c), the participants appeared to have gained more self- and disease insight and improved coping strategies. (d) to handle the challenges coming with the disease, including sexual challenges. For instance, one of the women described that she paid much attention to generally structuring her life after her diagnosis with BD, including her work life and social life and not engaging in random sexual relations that would destabilize her.

## Discussion

The purpose of this pilot study was to investigate BD and sexual health through in-depth interviews with individuals with BD. To our knowledge, this is the first study in the area presenting qualitative data, and we find it important to report our findings despite the small sample size, as we find the themes and the information from the participants very interesting. Further, we recognize the findings from the daily clinical experience when working with patients with BD. The preliminary study shows that the five participants experienced a connection between their bipolar episodes and changes in sexual drive, with increased sexual drive during manic episodes and lower and/or negative sexual drive during depressed episodes. Furthermore, changes in sexual interest seemed to act as an early warning sign of new episodes. We also wanted to explore whether feelings of shame were connected to their sexuality. However, from these interviews this seemed not to be the case. Our findings are consistent with findings from the few other studies that have examined how mood changes influence sexual function in individuals with BD (Kopeykina et al. 2016).

In this pilot study we also found that there may be gender differences in the ways in which BD and sexuality are related. During depressive phases, the men in the study experienced a destructive sex drive with negative feelings and thoughts associated with sex, rather than merely a reduced sex drive. However, due to the low number of participants, the data on this subject should be interpreted with caution. In UD, increased libido, especially in men, has been described in several articles (Laurent and Simons 2009). However, some argue that rather than an actual increased sexual desire it might be a search for reassurance and comfort (Laurent and Simons 2009). Regarding BD depression, a case report from Mahadevan et al. on this topic has been published, describing how a 48-year-old man with BD type II surprisingly had a higher sex drive and activity during his depressions (Mahadevan et al. 2013). A retrospective study from 2021 focused on adolescents who were hospitalized for UD or BD depression and examined different symptoms for the two kinds of depression (Meter et al. 2021). The study found that hypersexuality was significantly elevated in adolescents with BD depression compared to UD (Meter et al. 2021). We suggest further research on increased libido as a symptom of bipolar depression, and particularly the nature as well as the emotional and relational impact of this libido.

The clinical experience is often that “manic behavior” leads to feelings like shame and regret when returning to the euthymic state. In this study, however, none of the participants expressed feelings of shame about sexual escapades during hypomanic or manic phases. Placing their behavior in the realm of the natural and morally acceptable (“normal”) may be a coping strategy. In general Denmark is a liberal country concerning sexuality, which means that it is often natural and easy to speak about sexuality and having different partners and short-lived relationships. This also means that the findings from our five participants may not be correspond to findings in other cultural setting. On the other hand, it may still be difficult for the patients and health care providers to address sexual health, despite a relative liberal attitude in the society. It is also possible that positive feelings linked to sexual relations during manias overrule negative feelings of shame in depressed episodes. These observations are in line with a study by Fletcher et al. from 2012 (Fletcher et al. 2013) investigating 93 individuals with BD type II and their engagement in high-risk behavior, including being sexually disinhibited, and whether they thought their highs could be controlled or needed to be treated. The study found that risk behavior during hypomania was common and often had serious consequences, but less than one fifth of participants agreed that hypomania should be treated because of the associated risks. The patients romanticized their highs, which they found enjoyable. The same explanation might be transferable to our sample.

All participants described having had a high sex drive and experimental sexual behavior in their early youth and before diagnosis. A meta-analysis from 2016 by Van Meter et al. (Meter et al. 2016) looked at the prevalence of symptoms before an initial mood episode of BD. They included 11 studies and 1.078 men and women with an average age of 34.1 ± 8.9 years and found that hypersexuality was an “uncommon” prodrome symptom, meaning that it was reported by less than 25%, and that symptoms such as “too much energy” and “talkative” were presented by more than 50% (Meter et al. 2016). However, it still seems relevant to pay attention to increased sexual interest in young people where a diagnosis of BD is suspected.

Another objective of the study was to examine if individuals with BD requested sexological treatment in addition to their usual counseling. The interviews cast doubts on whether the participants found this relevant. Overall, it depended on whether the participants found their sexuality problematic. There was a tendency that sexual issues affected those who were in a relationship more than those who were single. Sexual problems could lead to problems in the relationship and be a burden to the partner. Along this line, a study by Lam et al. interviewing 37 partners of individuals with BD found that there were significant differences in sexual satisfaction during manic, depressed, and euthymic periods. The sexual satisfaction was lower for the partners when their partner with BD was either manic or depressed compared to well and there was no significant difference during the manic and depressed states (Lam et al. 2005). In general, during affective episodes the partners were less satisfied in the relationship, but no casual association between dissatisfaction with sex life and dissatisfaction with the relationship was seen (Lam et al. 2005). It seems tough highly relevant to focus on problems with relations, including (but not exclusively) sexual problems, in clinical practice and future research, as both may be affected by BD.

The participants had all participated in psychoeducation as part of the standard treatment at the clinic. Psychoeducation as an add-on treatment has several benefits, including reducing relapse rate, improving treatment adherence, improving social functioning and promoting healthy habits in patients (Duval et al. 2022; Bond and Anderson 2015). We found in our study that age and time living with a diagnosis of BD had improved coping strategies regarding sexuality and BD. However, one can speculate that psychoeducation also impacts the participants' sexual behaviour during episodes, even though it was not assessed in the interviews. For instance, one of the younger women, who only had a diagnosis of BD for one year, told how she have learned to canalize her increased energy into creative things when she was manic.

Sexual side effects related to psychotropic medication are often focused on, but this was not addressed directly in the interviews. However, both males mentioned that they had had problems reaching orgasm while on a specific medication, and one woman knew that her night medicine made her tired, which was a logistic consideration when wanting to have sex. All participants were prescribed mood stabilizers which is the first-line treatment for individuals with BD with a low risk for sexual side effects (Bowden et al. 2004). Four were prescribed low dose quetiapine, for which sexual side effects are also classified as non-common, which might be the reason medical sexual side effects might not have affected the participants.

## Limitations

This is a preliminary study, and with only five participants the transferability of the findings might be limited, although qualitative research methods aim for analytical, not statistical, transferability of the results. The point is to produce in-depth knowledge of participants’ experiences, rather than, for instance, quantifiable prevalence of symptoms. Still, interviews were not carried out until the data saturation point and for the results to be more generalizable, they need to be confirmed in a larger group of participants. A larger sample of patients would allow for subgroup analysis, including gender differences and the type of BD. For instance, a study found higher sexual interest in women with BD I compared to BD II and healthy controls (Mazza et al. 2011). Such analysis was not possible in our sample. Further, we could also have aimed for a more homogenous group, which would have strengthened our findings. The interviewer/interviewee interaction also affected how the interviews turned out. In our study the interview guide and the way the questions were asked reflected a culturally normative view on sexuality, with shame being one of the focus points, which may have inadvertently affected participants’ responses. Furthermore, although the intention was an interview with open questions, some closed questions were asked of the participants, which may also have limited the answers and insight into the topic. Caution should be taken when framing questions about sexual issues to avoid the risk of biasing responses.

Finally, it is possible that the five participants who did chose to participate (out of eight invited) were more open to talking about their sexual life than it is generally the case. Whether this openness also pertains to their sexual impulses and behavior is uncertain.

## Conclusions

This study is, to our knowledge, the first interview-based study describing individuals with BD’s perceptions of their sexuality. Our study was preliminary, including only five participants, the data should thus be interpreted cautiously. We found that changes in sexual behavior and drive were related to their affective episodes and vice versa. In the men, rather than reduced sexual drive, a negative sex drive prevailed in depressed episodes. We found no major feelings of shame related to hypersexual behavior during hypomanic and manic stages. Even though the participants doubted the necessity of implementing individual sexual counseling in addition to the usual clinical treatment, it does seem relevant from a clinical point of view. The study found a clear connection between sexuality and changes in mood and health-related quality of life in the individuals with BD. These findings need to be examined in a larger sample. We suggest focusing on shifts in sexual drive and a generally higher sex drive compared to peers during assessment in young individuals at increased risk of BD.

## Data Availability

The qualitative interview data used and analyzed during the current study are not publicly available due to the anonymity of the participants but are available from the corresponding author on reasonable request and with permission from the participant.
